# Extreme Neuroplasticity of Hippocampal CA1 Pyramidal Neurons in Hibernating Mammalian Species

**DOI:** 10.3389/fnana.2019.00009

**Published:** 2019-02-13

**Authors:** John M. Horowitz, Barbara A. Horwitz

**Affiliations:** Department of Neurobiology, Physiology and Behavior, University of California, Davis, Davis, CA, United States

**Keywords:** hippocampus, neuroplasticity, hibernation, memory, pyramidal cells (PC), LTP

## Abstract

In awake and behaving mammals (with core and brain temperatures at ~37°C), hippocampal neurons have anatomical and physiological properties that support formation of memories. However, studies of hibernating mammalian species suggest that as hippocampal temperature falls to values below ~10°C, CA1 neurons lose their ability to generate long term potentiation (LTP), a basic form of neuroplasticity. That is, the persistent increase in CA3-CA1 synaptic strength following high-frequency stimulation of CA3 fibers (the hallmark of LTP generation at 37°C) is no longer observed at low brain temperatures although the neurons retain their ability to generate action potentials. In this review, we examine the relationship of LTP to recently observed CA1 structural changes in pyramidal neurons during the hibernation cycle, including the reversible formation of hyperphosphorylated tau. While CA1 neurons appear to be stripped of their ability to generate LTP at low temperatures, their ability to still generate action potentials is consistent with the longstanding proposal that they have projections to neural circuits controlling arousal state throughout the hibernation cycle. Recent anatomical studies significantly refine and extend previous studies of cellular plasticity and arousal state and suggest experiments that further delineate the mechanisms underlying the extreme plasticity of these CA1 neurons.

## Converging Cellular Studies on the CA3-CA1 Synapse of CA1 Pyramidal Neurons

**Figure 1:**
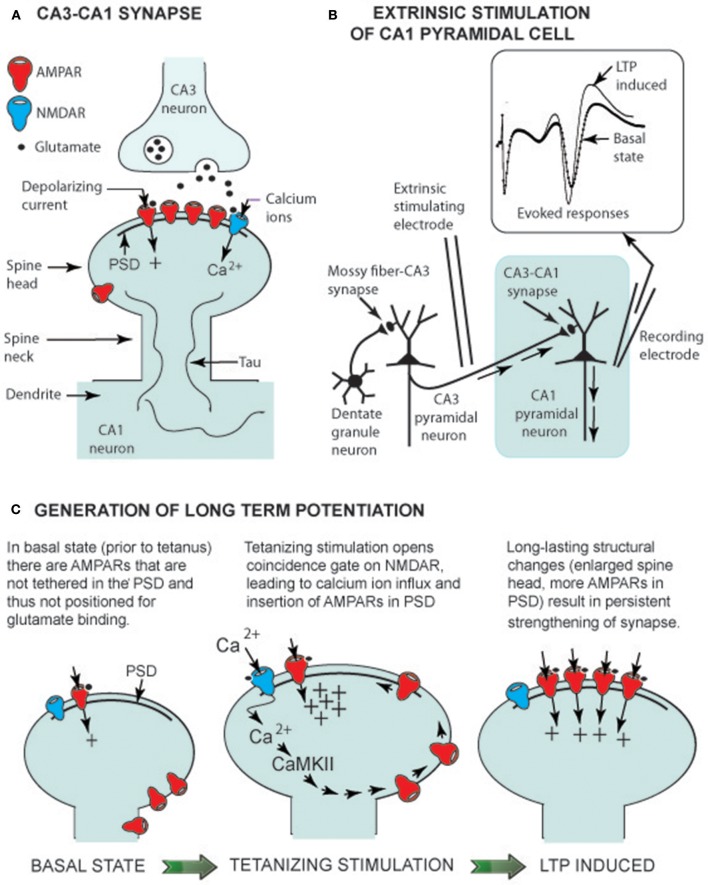
LTP generation in euthermic animals. **(A)** CA3-CA1 synaptic structure showing glutamate receptors (AMPARs and an NMDAR) linked to the post-synaptic density (PSD), a multiprotein assembly that orients receptors to face the presynaptic CA3 terminal. Tau is a structural protein that is not highly phosphorylated in the euthermic animal (see text). **(B)** Electrical circuit for recording CA1 pyramidal neuron-evoked responses. Insert shows evoked response prior to a tetanizing stimulation (basal state) and an enhanced response following the stimulation (LTP-induced). **(C)** Change in spine head shape before and after tetanizing stimulation. The latter induces a rapid (within seconds) increase in spine head size, allowing insertion of AMPARs into the PSD. Within minutes, the spine head has slightly shrunken to a long lasting (hours) form with additional AMPARs in the PSD (LTP-induced).

In hibernating mammals, two areas of research on hippocampal neurons have provided morphological and electrophysiological cellular data related to memory formation, a major function of the mammalian hippocampus. The morphological studies are built on observations that Golgi stained CA3 pyramidal neurons in Siberian ground squirrels (*Citellus undulates*) are smaller in winter when the squirrels are in torpor than in summer when they don't hibernate (Popov and Bocharova, 1992; Popov et al., 1992). These classic studies also showed that compared with neuron structure in summer, in torpor the neurons' apical dendrites had decreased length, decreased branching, and fewer spines. [Spines, mushroom shaped protuberances on dendrites, are the post-synaptic elements of many synapses (Figure 1A), and spine loss corresponds to a reduction in neural network connectivity.] Since these pioneering studies, others (e.g., Bullmann et al., 2016) have shown that in torpor, hippocampal CA1 pyramidal neurons display morphological retraction and spine loss as do CA3 pyramidal neurons.

A second group of studies involves neuroplasticity mechanisms at the synapse between a presynaptic CA3 axon branch (a Schaffer collateral) and a post-synaptic spine on a CA1 pyramidal neuron dendrite—i.e., the CA3-CA1 synapse (Figure 1A). In non-hibernating Syrian hamsters (*Mesocricetus auratus*), a form of neuroplasticity that strengthened synaptic signaling, long term potentiation (LTP; Figures 1B,C), was shown to be generated at the CA3-CA1 synapse at 22°C, but not at 20°C, even though at 20°C, stimulation of CA3 fibers still evoked action potentials in CA1 pyramidal neurons (Krelstein et al., 1990). Studies from Ingleman's lab further showed that LTP could be generated at 22°C in slices from Turkish hamsters (*Mesocricetus brandti*) in hibernation (Spangenberger et al., 1995). Since the 1990s, research on neuron morphology and neuroplasticity mechanisms in hibernating mammals has continued. However, until recently, species differences left “gaps” in both areas, limiting their merging into a more complete description of plasticity at CA3-CA1 synapses on CA1 pyramidal neurons as temperature falls and the animal enters hibernation.

These gaps were filled by two recent studies on Syrian hamsters—i.e., a major morphological study describing principal hippocampal neurons, including CA1 pyramidal neurons and their spines (Bullmann et al., 2016), and an electrophysiological study that delineated further properties of CA3-CA1 signal transmission (Hamilton et al., 2017). Both studies provide data on CA3-CA1 synapses; and this mini-review examines how these two areas of research on hibernating mammalian species have converged. Additionally, it more completely characterizes plasticity of CA1 pyramidal neurons as brain temperature declines and the animal enters torpor.

## Subcortical Neurons in Hibernating Species Continue to Process Signals at Low Brain Temperatures

Neural activity level in euthermic hibernating species (where T_brain_ = ~37°C) is similar to that in non-hibernating mammalian species and much greater than that in mammalian hibernators in torpor (T_brain_ = ~5–6°C). As temperature declines and the animal enters hibernation, neuron firing rates decrease throughout the brain (Kilduff et al., 1982). The CNS controls this decrease and continues to regulate T_brain_ throughout torpor (Florant and Heller, 1977; Heller, 1979). At T_brain_ = ~5°C in the hippocampus, theta and gamma oscillations are muted, and neocortical activity is greatly reduced, with EEG recordings flattening to nearly straight lines (Chatfield and Lyman, 1954; Beckman and Stanton, 1982).

Firing rate reduction throughout the whole brain contributes to energy conservation, thereby helping the animal survive throughout winters where food is scarce (Heller, 1979; Carey et al., 2003). Despite reduction in neuronal firing rates, subcortical brain regions continue to function and maintain homeostasis; i.e., body temperature remains regulated by the hypothalamus, and cardiorespiratory systems remain regulated by brainstem nuclei. These regulatory systems continue to function effectively in deep torpor as shown by continual adjustment of the animal's respiratory rate, thereby maintaining cell viability throughout the animal. Additionally, even in deep torpor, “alarm” signals (e.g., loud sounds, rapid drops in ambient temperature) arouse the animal from hibernation. Thus, evolutionary adaptations support reconfigurations of brain activity in torpor that maintain subcortical regulation of homeostasis and the processing of alarm signals while silencing neocortical EEG activity and attenuating hippocampal synchronized EEG activity.

Additional adaptations that reconfigure neural processing in torpor vary from species to species. Animals, such as marmots and arctic ground squirrels will only hibernate during winter (species denoted as obligatory or seasonal hibernators) while animals, such as Syrian and Turkish hamsters will hibernate any time of the year if exposed to cold and a short light-dark cycle (facultative hibernators). CNS clocks play a dominant role in determining when obligatory hibernators mate (spring), prepare for hibernation by storing energy/gaining weight (summer), and hibernate when food is scarce (winter). In contrast, behavior in facultative hibernators depends on ambient environment (light cycles, temperature, food availability) regardless of season. That is, Syrian hamsters in a constant warm environment with ample food will not hibernate, but if transferred to a winter-like environment, they acclimate to the cold, short day environment and then enter hibernation. Perhaps by chance, the first hibernation studies on morphological changes of hippocampal pyramidal cells were on obligatory hibernators, while the first study on hippocampal LTP generation was on hamsters. This left open the possibility that morphological changes were adaptations limited to obligatory species. However, more recent studies are consistent with the proposal that the data on neuron morphology reflect changes that occur in both obligatory and facultative hibernating mammals (e.g., Arendt et al., 2003; Bullmann et al., 2016).

## Glutamatergic Neurons at Low Brain Temperatures Continue to Support Signal Transmission Over Neural Circuits in Hibernation

Ramón y Cajal was the first to propose that principal hippocampal neurons form a chain of excitatory neurons (granule cell → CA3 pyramidal cell → CA1 pyramidal cell). His proposal has been confirmed, and the excitatory neurotransmitter has been identified as glutamate. These neurons share basic synaptic properties common to glutamatergic neurons throughout the brain, including the hypothalamus and brainstem—i.e., glutamate released by presynaptic neurons diffuses across the synaptic cleft and binds to two types of glutamatergic receptors on the spine of the post-synaptic neuron (Figure 1A). When glutamate binds to an AMPA receptor (AMPAR), the gated receptor channel opens, and a depolarizing current enters the post-synaptic neuron, contributing to generation of an action potential—i.e., a basic role of AMPARs is support of signal transmission from one neuron to the next. However, it is the operation of NMDA receptors (NMDARs) that has drawn wide attention because when their gate is opened, Ca^2+^ enters the spine and serves as a second messenger, activating pathways within the spine. In hippocampal CA1 neurons, Ca^2+^ activates a plasticity pathway generating LTP at T_brain_ = ~37°C.

Some neuronal ion channels (e.g., TRP channels) only operate over a limited temperature range (Voets et al., 2004), raising the question of whether AMPARs and NMDARs continue to operate at the low T_brain_ of hibernating mammals. That AMPARs do so is obvious because brainstem cardiorespiratory controllers rely on glutamatergic neurons to maintain homeostasis in awake and in hibernating hamsters. That is, telemetry recordings of blood pressure in unrestrained Syrian hamsters directly confirm that the baroreflex operates to regulate systolic pressure at ~96 mm Hg in euthermic hamsters and at ~39 mm Hg during torpor (Horwitz et al., 2013). The first neuron on this reflex is a glutamatergic neuron that responds to pressure (baroreceptors in the aortic arch) and excites second order neurons in the nucleus tractus solitarious (NTS), a brainstem nucleus.

The baroreceptor-second order NTS neuron synapse is an exemplar of a glutamatergic neuron that supports signal transmission throughout a hibernation cycle. Properties of Syrian hamster's AMPARs and NMDARs have been delineated at this synapse using patch-clamp techniques (Sekizawa et al., 2013). At both 33 and 15°C, glutamate binding to AMPARs gated their channels, allowing depolarizing ion currents to enter the cell, thus supporting signal transmission. Notably, NMDARs also remained functional at 33 and 15°C, and, when gated, Ca^+2^ entered the post-synaptic neuron. This gating required two simultaneous signals: neuron depolarization and glutamatergic binding to the receptor, a “coincidence gate” (Ascher and Nowak, 1988; Ascher et al., 1988).

Patch-clamp techniques have been used to directly control transmembrane potentials in *in vitro* slice preparations, thus demonstrating fully functional coincidence gating at 15°C and at 33°C. However, *in vivo*, firing rates of neurons are low during torpor, often resulting in cell depolarization that is insufficient to gate NMDARs. In contrast, because AMPARs are gated solely by glutamate binding (and are independent of cell depolarization), AMPARs maintain support of signal transmission from one neuron to the next.

## Hippocampal Plasticity

Two glutamatergic synapses in the hippocampus (Figure 1A), the mossy fiber–CA3 synapse and the CA3-CA1 synapse, are well-studied models of cellular neuroplasticity. LTP at the mossy fiber-CA3 pyramidal cell doesn't depend on NMDARs, but is entirely dependent on presynaptic modifications (Nicoll and Schmitz, 2005). In contrast, LTP at the CA3-CA1 synapse depends on glutamate gating NMDARs and post-synaptic spine modifications (Nicoll, 2017). In both hibernating and non-hibernating mammals, it is the CA3-CA1 synapse that has been most intensively studied. As Nicoll stated in his hippocampal plasticity review (2017), it is LTP at CA3-CA1 synapses that “holds the fascination of those working in this field because it provides a simple explanation for associative memory”.

Sustained potentiation of CA1 pyramidal cells observed following tetanus of Schaffer collaterals (Figure 1B), the defining property of LTP generation, has been observed in Syrian hamsters (Krelstein et al., 1990), Turkish hamsters (Spangenberger et al., 1995), and Yakutian ground squirrels (Pakhotin et al., 1990). Moreover, at T_brain_ = ~37°C, theta and gamma EEG oscillations provide an environment where Ca^2+^ entry into spines can activate cellular pathways. These data imply that NMDAR generation of LTP at the CA3-CA1 synapse in euthermic mammalian hibernating species is the same multistep process as in non-hibernating mammalian species.

In studies on the latter, the first step in NMDAR LTP generation is Ca^2+^ influx through NMDARs into spines. Once inside a spine, Ca^2+^ activates Ca^2+^ calmodulin-dependent protein kinase (CaMKII)—the initial step on a pathway leading to tethering of additional AMPARs to the PSD (a multiprotein assembly that orients receptors; see Figure 1C). As a result, the synapse is “strengthened” as additional AMPA receptors render the post-synaptic CA1 pyramidal cell more responsive to glutamate released from presynaptic Schaffer collaterals. The CaMKII–AMPAR pathway lies within the mushroom-shaped spine head, and the thin spine neck restricts diffusion of molecules to adjacent regions of the dendrite (Figure 1C). With this compartmentalization, glutamate release at a single synapse can selectively strengthen that synapse without influencing neighboring synapses (Hill and Zito, 2013).

Spines can readily change shape. Advances in imaging technology permit time-lapse observation of spine morphology in living neurons using fluorescent dyes and confocal or two-photon laser scanning microscopy. Over a timescale of hours, a spine can be seen to appear (bud out of a dendrite), grow to its signature mushroom shape, and then, hours to days later, retract and disappear back into the dendrite. Moreover, spines change shape within minutes when NMDA LTP is generated. That is, LTP-inducing stimuli result in an overshoot in spine size followed by a long-lasting spine enlargement (Figure 1C). This physical enlargement potentially enables the synapse to accommodate more AMPARs. Having reviewed studies on transient spine enlargement allowing added AMPAR insertion into the PSD, Herring and Nicoll (2016) concluded that LTP generation and morphological changes in spine structure go together in lockstep order.

The interlocking relationship of LTP generation and changes in spine shape is further illustrated by the finding that latrunculin A, an inhibitor of actin polymerization, not only blocked spine enlargement but LTP generation as well (Matsuzaki et al., 2004). A study on rat spine stabilization (Hill and Zito, 2013) showed that LTP generation had an effect on spine structure—i.e., LTP-inducing stimuli increased long-term survivorship (>14 h) of individual spines. Hill and Zito (2013) proposed that LTP-inducing stimuli promote transition of a new spine from a short-lived state (a retraction of the spine back into the dendrite), to a persistent state (a spine with maintained mushroom shape).

## A CA1 Pyramidal Cell Model for Small Mammalian Hibernating Species

**Figure 2:**
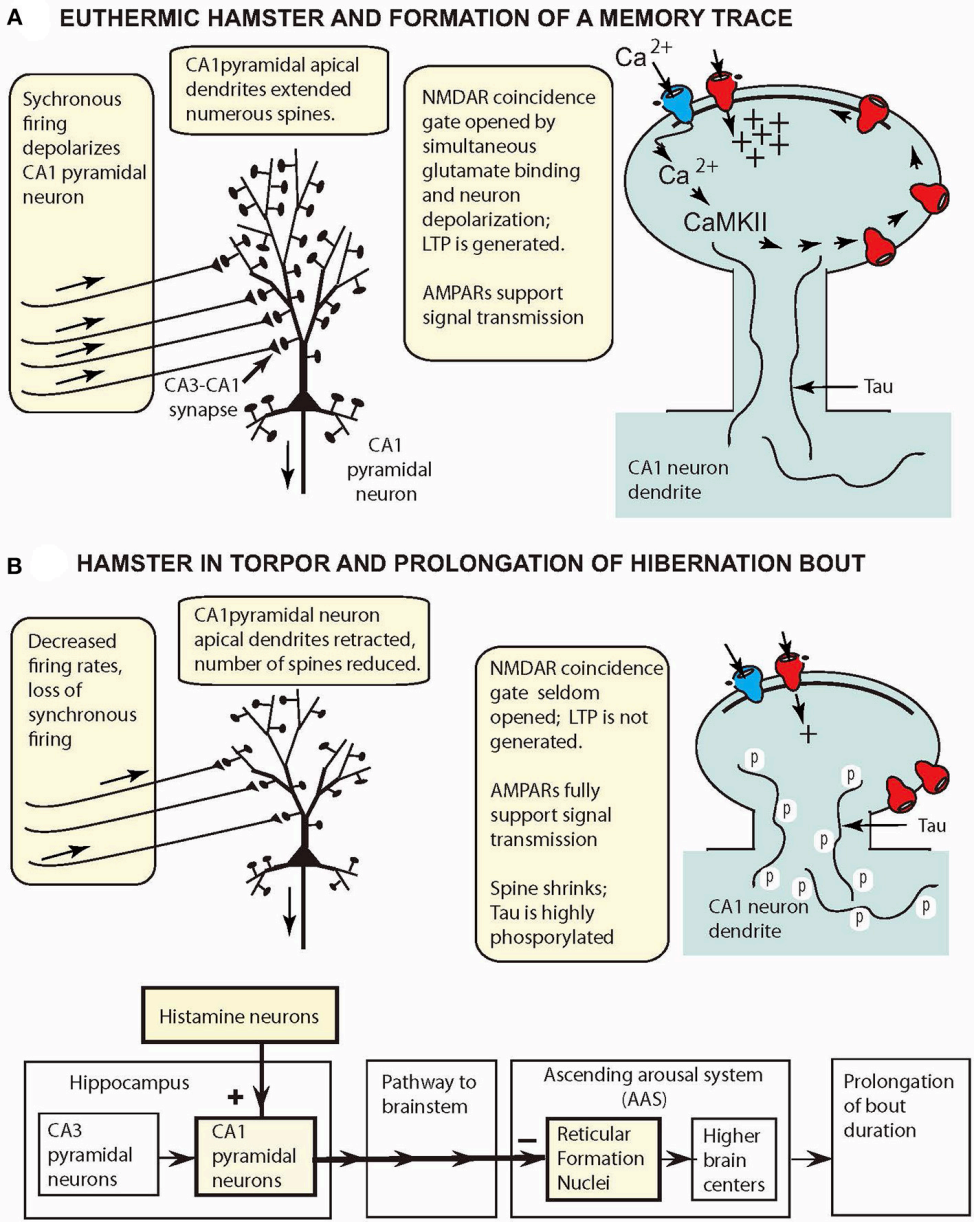
CA1 pyramidal cell model for small hibernating mammals (e.g., hamsters, ground squirrels), displaying key reversible adaptations when animal is **(A)** awake and **(B)** in torpor. **(A)** At 37°C, oscillatory hippocampal activity (theta and gamma waves) reflects synchronous excitation of CA1 pyramidal neurons (aligned arrows over afferent fibers). Coincidence gating of NMDARs leads to insertion of more AMPARs in the PSD and synapse strengthening. **(B)** In torpor, oscillatory activity is attenuated, and intrinsic activity fails to depolarize CA1 pyramidal neurons sufficiently to gate NMDARs. CA1 pyramidal neurons are retracted, spines are reduced in number, and tau is highly phosphorylated. These reconfigured neurons support signal transmission (via AMPARs) from the hippocampus to reticular formation nuclei to prolong hibernation bouts.

Combining Syrian hamster neuron morphology data (Bullmann et al., 2016) with LTP generation data (Hamilton et al., 2017) leads to the model for CA1 hippocampal pyramidal neurons depicted in Figure 2. Additional studies on obligatory species [e.g., on LTP (Pakhotin et al., 1990) and on morphology (Popov et al., 1992; Arendt et al., 2003)] extend this model to cover both obligatory and facultative hibernators.

At T_brain_ = ~37°C (Figure 2A), CA1 pyramidal neurons have long apical dendrites with numerous spines that support NMDAR LTP generation and AMPAR signal transmission, and the neurons appear to function in the same fashion as such neurons in non-hibernating mammalian species. However, in torpor (Figure 2B), extreme plasticity remodels the CA1 pyramidal neuron anatomically and physiologically. Highly phosphorylated tau in torpor (36–48 h of inactivity) is correlated with pyramidal cell retraction and reduction in the number of dendritic spines. Thus, in torpor, phosphorylated tau provides a marker of anatomical plasticity, a *natural reshaping* of the neuron into a smaller, compact form that requires less energy. These morphological changes are reversed upon arousal. Additionally, although NMDAR LTP is silenced in torpor, signal transmission via AMPARs is maintained, and hippocampal pyramidal neurons, like glutamatergic hypothalamic and brainstem neurons, continue to support signal transmission to other brain regions while minimizing energy consumption.

The model in Figure 2 can be easily augmented to incorporate additional neural properties. For example, the finding that in torpor, neurons in facultative and obligatory species have adaptations increasing their tolerance to oxygen-glucose deprivation (Mikhailova et al., 2016; Bhowmick et al., 2017) could be added to the figure.

## Consequences of Extreme Hippocampal Plasticity

A topic that has attracted continuing attention in hibernation studies is identification of brain regions controlling entrance into torpor, duration of torpor, and arousal from torpor. Beckman and Stanton (1982) consolidated early data suggesting that in torpor, the hippocampus sends signals over an inhibitory pathway to the brainstem reticular formation, resulting in prolongation of a hibernation bout. Their model built on the proposal that the reticular formation not only regulates waking and sleep as in non-hibernating mammalian species (Moruzzi and Magoun, 1949; Fuller et al., 2011), but has adaptations in hibernators that extend the arousal system to a continuum of distinct behavior states: waking, sleep, and hibernation. Additional *in vivo* studies showed that bilateral infusion of histamine into hippocampi of hibernating ground squirrels increased bout duration (Sallmen et al., 2003), and *in vitro* slice studies showed that histamine altered hamster CA1 pyramidal cell excitability (Nikmanesh et al., 1996; Hamilton et al., 2017). The CA1 pyramidal cell model has exactly the properties needed for CA1 pyramidal cells to take on a new role in torpor and process signals prolonging bout duration (Figure 2B). Future experiments are needed to precisely delineate the anatomical pathway from the hippocampus to the arousal system, experiments now feasible because major nuclei in the ascending arousal system have been identified (Fuller et al., 2011; Pedersen et al., 2017).

A second topic that has attracted attention focuses on whether memories formed in euthermic hamsters are erased in torpor as neurons retract and spines vanish back into dendrites. Behavioral studies provide mixed results depending on species, animal behavior, and experimental design (Bullmann et al., 2016). For example, European ground squirrels (*Spermophilus citellus*) that learned a spatial memory task in summer, hibernated in winter, and when retested the following spring, showed clear impairment in performance compared with controls [squirrels kept in a warm environment during winter (Millesi et al., 2001)]. In contrast, Bullmann et al. (2016) showed that Syrian hamsters that had mastered a hippocampal maze task in a summer-like environment and were retested following a series of torpor bouts had no impairment in performance. This memory retention in hamsters is likely due to a variety of adaptations (Bullmann et al., 2016). Additionally, LTP-inducing stimuli may have promoted transition of spines from a short-lived to a persistent state (Hill and Zito, 2013) such that surviving spines encoded earlier memories. The relationship of changes in neuron configuration to behavior is further illustrated by the finding in Arctic ground squirrels (*Spermophilus parryii)* that contextual learning and memory was altered for a few days following arousal as neurons overshot in size and then returned to a pre-hibernation configuration (Weltzin et al., 2006). Future behavioral experiments are needed to more completely characterize the cellular properties that support the remarkable memory retention of Syrian hamsters.

Can new memories be formed while the hamster is in torpor? Since *in vitro* experiments show that LTP is arrested at T_slice_ ≤ ~15°C, it appears unlikely that pyramidal cells can effectively contribute to formation of new memories during torpor (Hamilton et al., 2017). Moreover, in torpor, tau is highly phosphorylated, apical dendrites are retracted, and the number of spines on dendrites are reduced (Bullmann et al., 2016)—all evidence suggesting that neurons in torpor are not as well-configured to form new memories as they are in euthermic hamsters. Additionally, although *in vitro* slice preparations permit extrinsic stimulation (repeated bursts of shocks) to Shaffer collaterals at all slice temperatures, *in vivo* studies show no equivalent intrinsic stimulatory signal as oscillatory EEG activity is attenuated in torpor (Chatfield and Lyman, 1954). Thus, because in mammals at T_brain_ = ~37°C, hippocampal gamma and theta oscillations play a natural role in LTP induction *in vivo* (Bikbaev and Manahan-Vaughan, 2008), attenuation of oscillatory activity suggests LTP cannot be induced when T_brain_ = ~5°C.

## Summary

CA1 pyramidal neurons in euthermic hamsters (and other small hibernating species) are configured to support formation of memory traces (Figure 2A). But it is the natural adaptations that reconfigure CA1 pyramidal neurons in torpor (Figure 2B) that have drawn attention of workers in the field. Low levels of neural activity suspend NMDAR LTP generation in torpor. Despite neuron retraction and spine loss during torpor, memory retention of tasks learned prior to torpor and retested after torpor is moderate in ground squirrels and excellent in hamsters. A notable feature of the compact CA1 neurons in torpor is that they are able to conserve energy and support signal transmission via AMPARs. Thus, they appear to be well-configured to prolong hibernation bouts, and if so, would strengthen the proposal that the hippocampus joins other brain regions in contributing to the neural control of hibernation.

## Author Contributions

Both authors have made a substantial, direct and intellectual contribution to the work, and approved it for publication.

### Conflict of Interest Statement

The authors declare that the research was conducted in the absence of any commercial or financial relationships that could be construed as a potential conflict of interest.
